# Structures and electronic properties of cobalt(II) selone coordination com­plexes

**DOI:** 10.1107/S2053229625010101

**Published:** 2025-11-26

**Authors:** Shaydel M. Purcell, Eric W. Reinheimer, Jamie S. Ritch

**Affiliations:** a Department of Chemistry, The University of Winnipeg, 515 Portage Ave, Winnipeg, MB R3B 2E9, Canada; b Rigaku Americas, The Woodlands, Texas 77381, USA; University of Sydney, Australia

**Keywords:** seleno­urea ligand, cobalt(II) com­plex, coordination chemistry, crystal structure, density functional theory, thermochemistry

## Abstract

The structural chemistry of seleno­urea ligands is quite diverse, though examples of their coordination to cobalt are rare. In this study, the solid-state structures of the seleno­urea 1,3-di­ethyl­im­id­a­zole-2-selone, C_7_H_12_N_2_Se, and the cobalt com­plexes di­chlorido­bis­(1,3-di­ethyl­im­id­a­zole-2-selone-κ*Se*)cobalt(II), [CoCl_2_(C_7_H_12_N_2_Se)_2_] (**1**), and di­chlorido­bis­(1,3-diiso­propyl­im­id­a­zole-2-selone-κ*Se*)cobalt(II), [CoCl_2_(C_9_H_16_N_2_Se)_2_] (**2**), are presented. Two crystallization methods for the coordination com­plexes are utilized. The structures of com­plexes **1** and **2** are com­pared with the few existing examples in the literature, revealing a similar trend for terminal binding modes, rather than bridging modes which are often seen for late *d*-block metal com­plexes. Density functional theory calculations reveal a trend in cobalt–selenium bond strengths for 1,3-dialkyl-substituted im­id­a­zole-2-selones of Me ≃ Et < ^i^Pr.

## Abstract 2

The structures of a cyclic seleno­urea ligand and two distorted tetra­hedral cobalt(II) chloride com­plexes are disclosed, along with their com­puted electronic properties and thermochemistry.

## Introduction

Cyclic seleno­ureas have a diverse and expansive coordination chemistry, serving as monodentate Se-centred ligands which can adopt terminal or bridging binding modes toward a variety of metals. The structural chemistry of late *d*-block metals, such as gold, copper and palladium, are particularly well-explored (Ritch, 2019 ▸). By contrast, examples of structurally-characterized first-row transition-metal com­plexes, such as those of cobalt, are much rarer. Early work on cobalt(II) seleno­urea chemistry involved IR and electronic spectral characterization on a series of thio- and seleno­urea com­plexes of cobalt(II) halides (Devillanova *et al.*, 1981 ▸), though no solid-state structures were presented. Since that study, only two examples of cyclic seleno­urea cobalt(II) com­plexes have been structurally characterized (Williams *et al.*, 1997 ▸; Jia *et al.*, 2008 ▸).

Cobalt coordination com­plexes are of increasing importance in the homogeneous catalysis of organic transformations (Li *et al.*, 2021 ▸), as a replacement for those based on rarer and more expensive second- and third-row metals. Also seeing rapid development are solid-state cobalt selenide phases with applications including electrocatalysis of water-splitting reactions (Zhang *et al.*, 2019 ▸). In this context, fundamental studies of the coordination chemistry of cobalt towards selenium-centred ligands will help increase our understanding of structure–activity relationships and aid in the rational design of new mol­ecules and materials. In this study, the crystal structures of one seleno­urea ligand and two cobalt(II) seleno­urea coordination com­plexes are reported (Scheme 1). Structural com­parisons to reported examples are presented, along with a com­putational analysis of ligand binding strengths and the conformational energetics of the com­plexes.

## Experimental

Syntheses were performed using standard techniques without any special precautions to exclude air or moisture. The reagents 1,3-di­ethyl­im­id­a­zolium iodide, 1,3-di­ethyl­im­id­a­zole-2-selone (deise) and 1,3-diiso­propyl­im­id­a­zole-2-selone (diise) were made by modifications of a literature procedure (Williams *et al.*, 1993 ▸). 1,3-Diiso­propyl­im­id­a­zolium chloride was prepared *via* the reported procedure of Schaub & Radius (2005 ▸). Other reagents and solvents were purchased from commercial sources and used as received. NMR spectra were recorded on a Bruker Avance III 400 MHz NMR spectrometer.
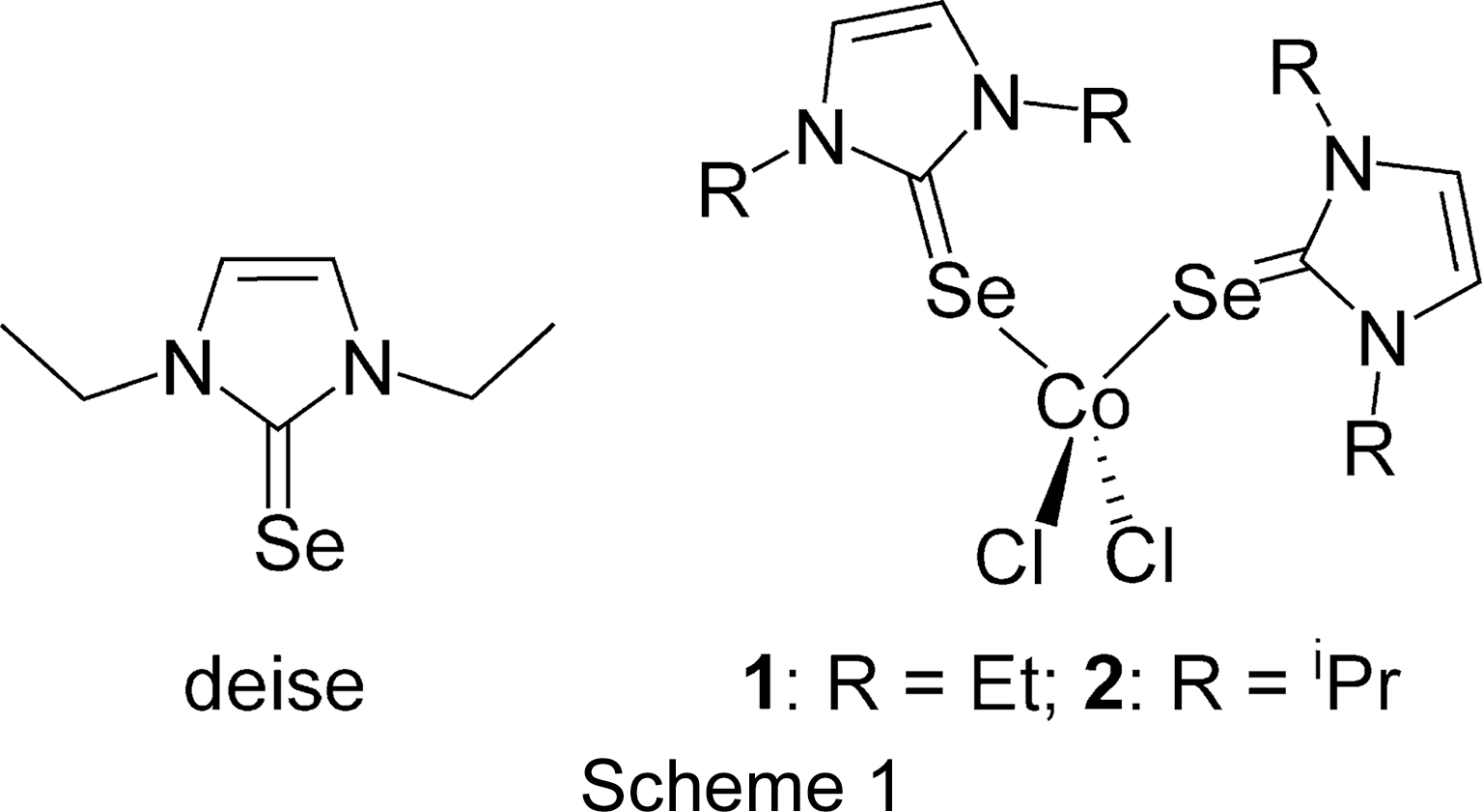


### Computational details

Geometry optimizations and frequency calculations were performed with the *ORCA* software package (Version 6.1.0; Neese, 2025 ▸) using the ωB97M-V functional (Mardirossian & Head-Gordon, 2016 ▸) and the def2-TZVP basis set (Weigend & Ahlrichs, 2005 ▸), with no symmetry constraints. Final single-point energies were com­puted at the wB97M-V/def2-QZVPP level. All calculations are gas phase, with no solvent corrections, and thermochemistry values are given for conditions of 298.15 K and 1 atm. Open-shell Co^II^ species were modelled using the unrestricted Kohn–Sham formalism with a quartet ground state. Relaxed potential energy scans were performed by optimizing the structures with the desired torsion angle constrained to values incremented from 0 to 180° in 10° steps. Natural atomic charges were evaluated using the *NBO7* software package (Glendening *et al.*, 2018 ▸).

### Synthesis and crystallization

#### Synthesis of deise

1,3-Di­ethyl­im­id­a­zolium iodide (3.479 g, 13.8 mmol), Se (2.7516 g, 34.8 mmol), K_2_CO_3_ (3.8150 g, 27.6 mmol) and methanol (40 ml) were added to a 250 ml round-bottomed flask, which was equipped with a reflux condenser and heated to reflux for 21 h. After cooling to room tem­per­a­ture, the volatiles were removed using a rotary evaporator. Di­chloro­methane (30 ml) was added and the mixture was filtered through a medium-porosity fritted funnel. The filtrate was concentrated on a rotary evaporator to remove most of the solvent and the resulting pale-yellow liquid was left to crystallize at −25 °C. The remaining liquid was deca­nted and the solid was dried in air, affording a colourless crystalline product (yield: 1.3765 g, 6.8 mmol, 49%). NMR spectral data matched those reported in the literature. X-ray-quality crystals were selected from the as-prepared product.

#### Synthesis of diise

To a 100 ml round-bottomed flask equipped with a stirrer bar was added 1,3-diiso­propyl­im­id­a­zolium chloride (251.2 mg, 1.33 mmol), Se (104.9 mg, 1.33 mmol), K_2_CO_3_ (223.1 mg, 1.61 mmol) and aceto­nitrile (50 ml). A reflux condenser was attached and the mixture was heated to reflux for 17 h. After cooling to room tem­per­a­ture, the volatiles were removed using a rotary evaporator. The resulting mixture was extracted with CH_2_Cl_2_ (25 ml) and filtered through a medium-porosity fritted funnel to afford a clear yellow solution. Removing the volatiles under vacuum and recrystallization from methanol at −25 °C afforded the product as colourless crystals (yield: 87.14 mg, 0.38 mmol, 28%). NMR spectral data matched those reported in the literature.

#### Synthesis of [CoCl_2_(deise)] (1)

CoCl_2_·6H_2_O (204.6 mg, 0.50 mmol) was dissolved in triethyl orthoformate (2.5 ml) in a 10 ml round-bottomed flask. In a beaker, deise (204.6 mg, 1.0 mmol) was dissolved in triethyl orthoformate (2 ml) and di­chloro­methane (1 ml) with stirring, then added to the cobalt chloride solution. Immediate formation of a green suspension was observed. After stirring for 1 h, the product was isolated by vacuum filtration into a medium-porosity fritted funnel, washed with Et_2_O (2 × 3 ml) and dried under suction. The procedure yielded a green fine powder. X-ray-quality crystals were obtained by slow evaporation of a methanol solution of the com­plex at room tem­per­a­ture.

#### Synthesis of [CoCl_2_(diise)] (2)

The com­plex was prepared according to the method used for the preparation of the 1,3-di­methyl­im­id­a­zole-2-selone (dmise) analogue (Williams *et al.*, 1997 ▸). CoCl_2_·6H_2_O (45.0 mg, 0.19 mmol), diise (86.5 mg, 0.37 mmol) and me­tha­nol (15 ml) were added to a 50 ml round-bottomed flask and the resulting solution concentrated by boiling until green in colour. Cooling afforded green crystals and a yellow solution. Decanting the liquid, then washing the solid with iso­propanol (2 × 5 ml) and CHCl_3_ (2 × 3 ml) afforded the product as green X-ray-quality crystals.

### Refinement

Crystal data, data collection and structure refinement details are summarized in Table 1 ▸. H atoms were placed in calculated positions and refined according to a riding model [C—H = 0.97 Å and *U*_iso_(H) = 1.5*U*_eq_(C) for tetra­hedral carbon centres; C—H = 0.95 Å and *U*_iso_(H) = 1.2*U*_eq_(C) for trigonal planar carbon centres]. The structure of deise features disorder of one ethyl group. This was modelled as an anisotropic mixture of the terminal C atom over two positions in a 80:20 occupancy, as refined by a free variable. The two partially overlapping C-atom positions were additionally re­strain­ed to have similar *U^ij^* com­ponents.

## Results and discussion

The seleno­urea ligands diese and diise were used in this investigation. Preparations of both have been reported previously. Diese was prepared from the im­id­a­zolium bromide salt *via* deprotonation in the presence of elemental Se with either (i) K[N(SiMe_3_)_2_] at low tem­per­a­ture in tetra­hydro­furan (THF; Barnett *et al.*, 2021 ▸) or (ii) Na_2_CO_3_ in refluxing water (Huang *et al.*, 2021 ▸). Diise was prepared in a similar manner from selenium and the im­id­a­zolium tetra­fluoro­borate salt and KO^t^Bu in THF at room tem­per­a­ture (van Weerdenburg *et al.*, 2015 ▸) or from the bromide salt and Na[N(SiMe_3_)_2_] in THF at low tem­per­a­ture (Verlinden *et al.*, 2015 ▸). We have found that both deise and diise can be prepared in a simpler protocol similar to that reported for the synthesis of dmise (Williams *et al.*, 1993 ▸) using Se and K_2_CO_3_ and 1,3-dii­ethyl­im­id­a­zolium iodide or 1,3-diiso­propyl­im­id­a­zolium chloride in refluxing solvent (methanol for deise and aceto­nitrile for diise). These reactions can be performed in air without any special precautions to exclude moisture.

**Figure 1:**
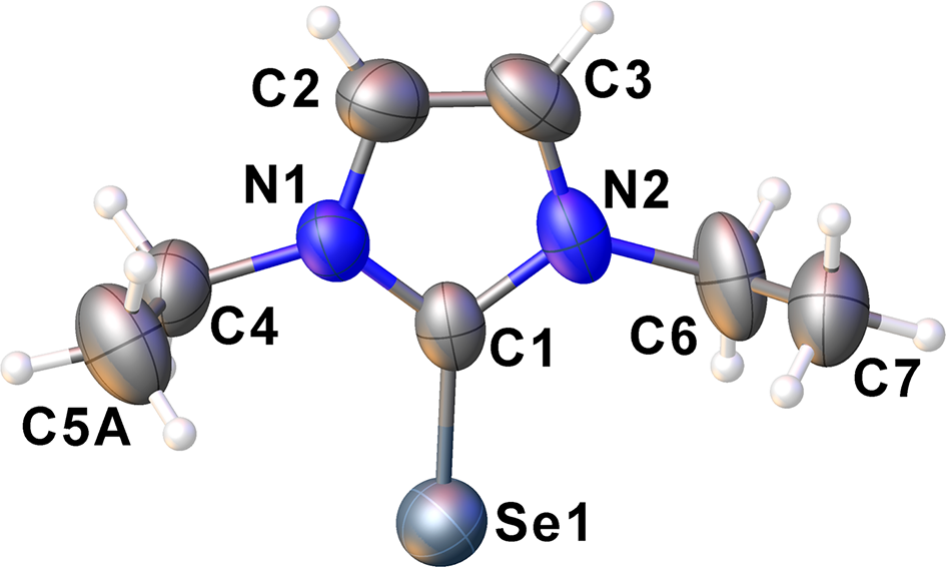
Displacement ellipsoid plot (50% probability level) of deise. Atom C5*A* is one part of a two-part anisotropic disorder model; the minor com­ponent has been omitted for clarity.

While the ligand diise has been structurally characterized (van Weerdenburg *et al.*, 2015 ▸), deise has not. The as-prepared crystals of deise were of X-ray quality and were determined to form in the space group *P*2_1_/*n*. A displacement ellipsoid plot of deise is shown in Fig. 1 ▸. The C=Se bond length of 1.835 (4) Å is not significantly different from the reported distances for dmise and diise. The ethyl groups are in a *syn* conformation, presumably to increase packing efficiency. Other metrical parameters are also unremarkable and there are no significant inter­molecular inter­actions.

The pseudo­tetra­hedral Co^II^ com­plex of dmise, *i.e.* [CoCl_2_(dmise)_2_], has been reported previously (Williams *et al.*, 1997 ▸). In that study, crystals were obtained by boiling down an ethano­lic solution of ligand and CoCl_2_·6H_2_O. A helical coordination polymer com­plex of 1,1′-methyl­enebis(3-methyl­im­id­a­zoline-2-selone) (mbis), also with distorted tetra­hedral coordination geometry, was made as a powder from CoCl_2_ and ligand in THF followed by recrystallization from CH_3_CN/Et_2_O (Jia *et al.*, 2008 ▸). To date, these represent the only structurally characterized com­plexes of cobalt(II) with a sel­eno­­urea ligand. Several octa­hedral Co^III^ com­plexes con­tain­ing seleno­urea ligands, SeC(NH_2_)_2_, have also been reported (Rija *et al.*, 2011 ▸).

We found two methods suitable for preparing cobalt(II) com­plexes of deise and diise. The method of Williams *et al.* (1997 ▸) was used for the diise com­plex, **2**, while for the deise com­plex, **1**, a method used for preparing an iron(II) com­plex of dmise was used (Stadelman *et al.*, 2016 ▸). In this procedure, a solvent mixture of triethyl orthoformate and di­chloro­methane is used, the former also acting as an *in-situ* dehydrating agent. The powdered product can be filtered out of the reaction mixture. The ethyl and isopropyl com­plexes are stable in crystalline form under ambient conditions (stored in a vial under air) for years. They can be recrystallized from concentrated solutions of methanol, though when dissolving in this solvent they give yellow solutions which evaporate to give crystals of both the free ligand and com­plex, indicating methanol is com­petitive with the ligands for coordination to cobalt(II).

**Figure 2:**
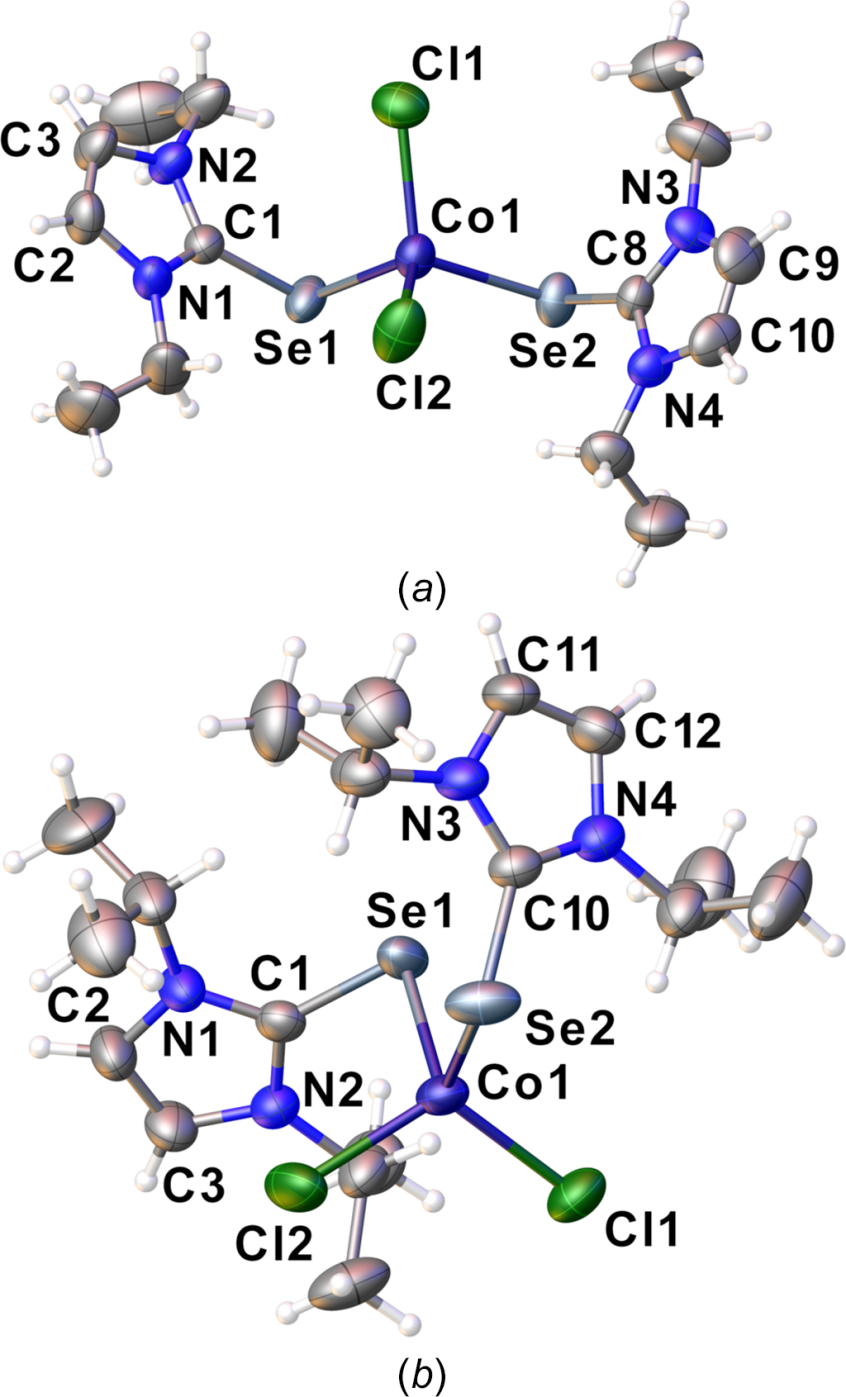
Displacement ellipsoid plots (50% probability level) of (*a*) com­plex **1** and (*b*) com­plex **2**.

Complexes **1** and **2** both crystallize from methanol in the space groups *P*2_1_/*c* and *P*2_1_/*n*, respectively, and their displacement ellipsoid plots are shown in Fig. 2 ▸. Each structure features one mol­ecule in the asymmetric unit. The Co—Se distances in both com­plexes are in the narrow range of 2.4622 (5)–2.4720 (7) Å, and are consistent with the distances reported for the dmise com­plex (Williams *et al.*, 1997 ▸). Likewise, the C=Se bond lengths in the com­plexes show a slight elongation of 1–2% in com­plexes **1** [1.879 (4)–1.883 (4) Å] and **2** [1.873 (3)–1.874 (3) Å] *versus* the free ligands deise [1.835 (4) Å] and diise [1.849 (2) Å], consistent with reports for the dmise com­plex.

While these com­plexes all share a common pseudo­tetra­hedral CoSe_2_Cl_2_ core, the most dramatic difference in their structures is the relative orientation of the two seleno­urea ligands. The com­plexes exhibit values of the pseudo-torsion angle τ(C=Se⋯Se=C) of 97.9 (5)° for [CoCl_2_(dmise)_2_], 36.8 (1)° for **1** and 144.4 (1)° for **2**. Notably, com­plexes **1** and **2**, as well as all other structurally determined cobalt–seleno­urea com­plexes, show only terminal coordination of the seleno­urea ligand, with no bridging μ-Se inter­actions. Seleno­urea com­plexes of late *d*-block metals, including Cu^I^, Ag^I^ and Pd^II^ have shown bridging in their solid-state structures *via* μ_2_-Se or μ_2_-halide motifs (Ritch, 2019 ▸).

**Figure 3:**
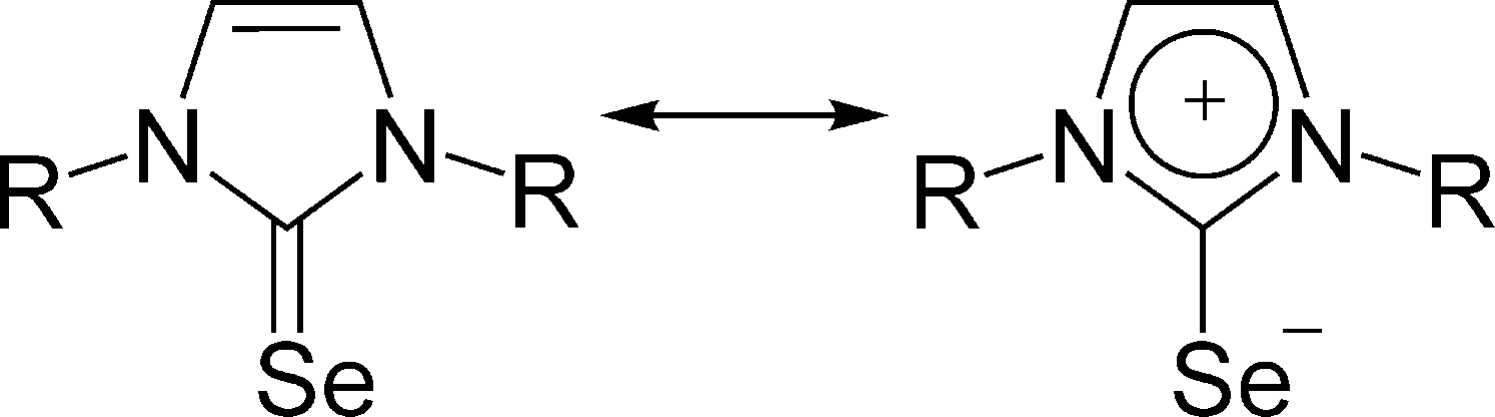
Resonance contributors to the structure of cyclic seleno­ureas.

Geometry optimizations of dmise, deise and diise, as well as their CoCl_2_*L*_2_ com­plexes, were conducted at the ωB97M-V/def2-TZVP level of theory. The free ligands show a trend of slightly increasing C=Se bond length as the size of the alkyl chain increases, spanning a 1% difference from dmise to diise (dmise: 1.828 Å; deise: 1.836 Å; diise: 1.847 Å). This trend is not reflected in the experimental values, which are all equal within error due to the size of the standard uncertainties. Considering the two resonance forms of seleno­urea ligands (Fig. 3 ▸), the com­puted distances indicate that more electron-releasing alkyl groups slightly favour the C—Se resonance structure, where there is less *p*(Se)→*p*(C) donation and hence less π bonding between these atoms. This is corroborated in the energies of the Se(*p*)-type highest occupied molecular orbitals (HOMOs), which increase in energy for *R* = Me (−7.314 eV) > *R* = Et (−7.264) > *R* = ^i^Pr (−7.186). Additionally, the atomic charges on the Se atoms obtained through natural population analysis show the trend *R* = Me (−0.31) < *R* = Et (−0.33) < *R* = ^i^Pr (−0.34).

To com­pare the ability of seleno­ureas to donate to a high-spin Co^II^ centre *versus* a more common phospho­rus-centred ligand, their putative metathetical reactions with [CoCl_2_(PPh_3_)_2_] to form [CoCl_2_(*L*)_2_] products [Equation (1)] were modelled at the ωB97M-V/def2-QZVPP level of theory. Gas-phase thermochemistry of the reactions is summarized in Table 2 ▸. The standard enthalpies of reaction for *R* = Me and Et are similar, being slightly exothermic, while *R* = ^i^Pr shows a significantly more exothermic reaction. Since there is very little geometric distortion of any of the ligands upon coordination or dissociation, the Δ*H*° values are explained by the seleno­ureas forming stronger bonds to Co^II^ than triphenylphosphine, particularly the isopropyl-substituted variant. This is in keeping with the HOMO energies and indicates that for Co^II^ the isopropyl seleno­urea is a significantly more strongly donating ligand than the smaller alkyl chain variants or even PPh_3_. The seleno­urea diise is thus expected to be a com­petent ligand for other low-valent transition metals.

[CoCl_2_(PPh_3_)_2_] + 2*L* → [CoCl_2_(*L*)_2_] + 2 PPh_3_ (1)

**Figure 4:**
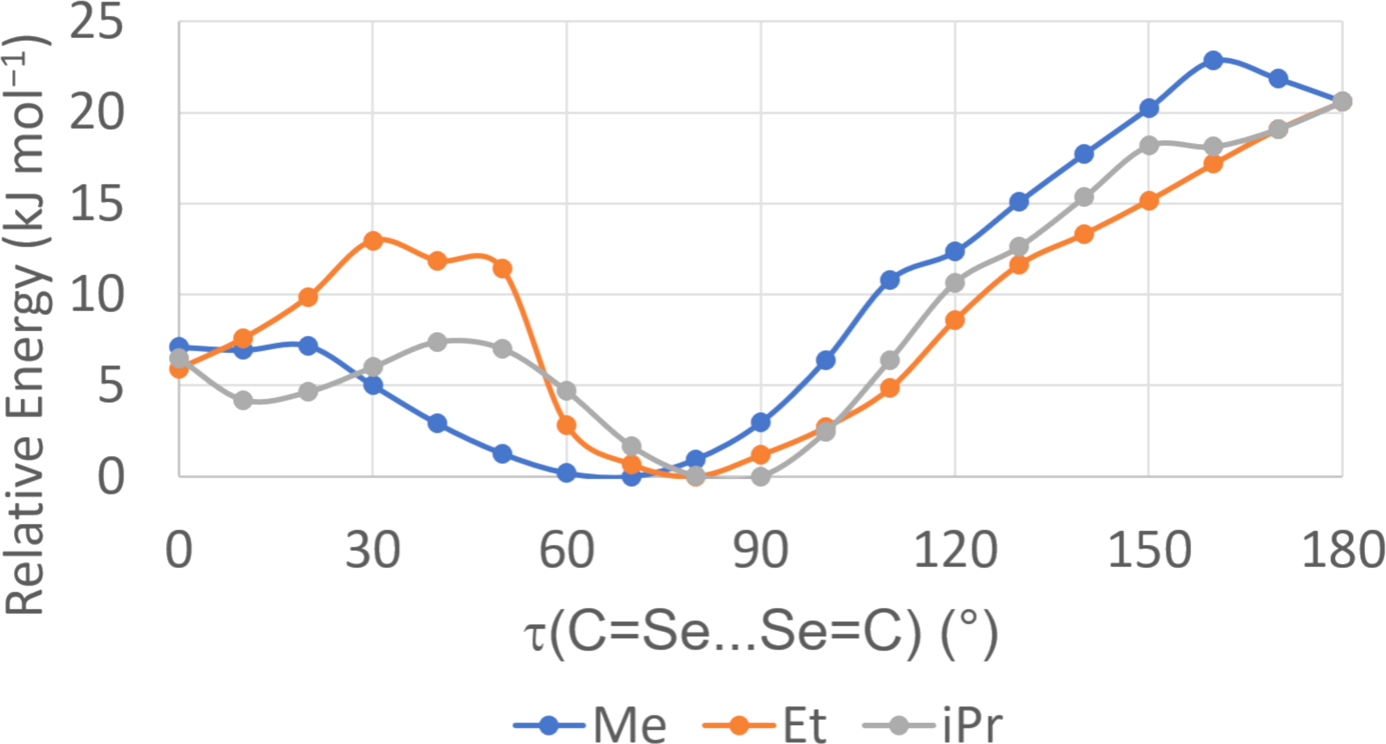
Conformation energy diagram for cobalt(II) com­plexes of seleno­ureas (dmise: *R* = Me; deise: *R* = Et; diise: *R* = ^i^Pr).

Given the wide range of τ(C=Se⋯Se=C) angles observed in the structures of CoCl_2_(*L*)_2_, conformational analyses were conducted on each com­plex by a relaxed potential energy surface scan of this angle from 0–180° (*i.e.* from synplanar to anti­planar). Very similar results were seen in each case (Fig. 4 ▸), with a potential energy minimum around 70–90° and an overall range in energies of *ca* 20–23 kJ mol^−1^, indicating a shallow potential energy surface about this angle. The differing conformers observed in the crystal structures are therefore not surprising and can be rationalized by varying packing forces in each case. In solution, a mixture of conformers can be reasonably expected.

The structures presented herein provide rare new examples of cobalt(II) coordination com­plexes of seleno­ureas. They exhibit conformational flexibility, as well as long-term stability in the solid state, though in polar protic solvents the com­plexes are labile. The alkyl-substituted seleno­urea ligands dmise, deise and diise are com­puted to have a stronger inter­action with Co^II^ than PPh_3_, particularly the isopropyl variant. This knowledge will be utilized in the design of future ligand iterations aimed at preparing com­plexes with increased solution-state stability.

## Supplementary Material

Crystal structure: contains datablock(s) deise, 1, 2, global. DOI: 10.1107/S2053229625010101/wv3022sup1.cif

Structure factors: contains datablock(s) deise. DOI: 10.1107/S2053229625010101/wv3022deisesup2.hkl

Structure factors: contains datablock(s) 1. DOI: 10.1107/S2053229625010101/wv30221sup3.hkl

Structure factors: contains datablock(s) 2. DOI: 10.1107/S2053229625010101/wv30222sup4.hkl

Supporting information file. DOI: 10.1107/S2053229625010101/wv3022deisesup5.cml

Atomic coordinates and energies of all DFT-computed structures (.xyz format). DOI: 10.1107/S2053229625010101/wv3022sup6.txt

CCDC references: 2502265, 2502264, 2502263

## Figures and Tables

**Table 1:** Experimental details For all structures: *Z* = 4. Experiments were carried out with Mo *K*α radiation. H-atom parameters were constrained.

	**deise**	**1**	**2**
Crystal data			
Chemical formula	C_7_H_12_N_2_Se	[CoCl_2_(C_7_H_12_N_2_Se)_2_]	[CoCl_2_(C_9_H_16_N_2_Se)_2_]
*M*_r_	203.15	536.12	592.22
Crystal system, space group	Monoclinic, *P*2_1_/*n*	Monoclinic, *P*2_1_/*c*	Monoclinic, *P*2_1_/*n*
Temperature (K)	293	293	302
*a*, *b*, *c* (Å)	6.8733 (4), 14.6494 (9), 9.0726 (5)	12.9700 (6), 12.1282 (6), 13.5313 (6)	10.1594 (2), 15.7281 (4), 15.8120 (4)
β (°)	94.493 (6)	92.715 (4)	91.000 (1)
*V* (Å^3^)	910.71 (9)	2126.12 (17)	2526.18 (10)
μ (mm^−1^)	4.06	4.49	3.78
Crystal size (mm)	0.32 × 0.29 × 0.27	0.36 × 0.27 × 0.1	0.20 × 0.08 × 0.05

Data collection			
Diffractometer	Rigaku XtaLAB Mini II	Rigaku XtaLAB Mini II	Bruker D8 QUEST ECO
Absorption correction	Analytical (*CrysAlis PRO*; Rigaku OD, 2025 ▸)	Analytical (*CrysAlis PRO*; Rigaku OD, 2025 ▸)	Multi-scan (*SADABS*; Bruker, 2016 ▸)
*T*_min_, *T*_max_	0.623, 0.685	0.420, 0.754	0.630, 0.746
No. of measured, independent and observed [*I* > 2σ(*I*)] reflections	5662, 1627, 1134	15089, 3747, 2710	61212, 5165, 3994
*R*_int_	0.024	0.032	0.038
(sin θ/λ)_max_ (Å^−1^)	0.598	0.595	0.625

Refinement			
*R*[*F*^2^ > 2σ(*F*^2^)], *wR*(*F*^2^), *S*	0.037, 0.074, 1.03	0.036, 0.070, 1.01	0.033, 0.084, 1.04
No. of reflections	1627	3747	5165
No. of parameters	104	212	252
No. of restraints	6	0	0
Δρ_max_, Δρ_min_ (e Å^−3^)	0.38, −0.41	1.07, −0.78	0.64, −0.33

Computer programs: *CrysAlis PRO* (Rigaku OD, 2025 ▸), *SAINT* (Bruker, 2016 ▸), *APEX3* (Bruker, 2016 ▸), *SHELXT2014* and *SHELXT2018* (Sheldrick, 2015*a* ▸), *SHELXL2016* and *SHELXL2018* (Sheldrick, 2015*b* ▸), and *OLEX2* (Dolomanov *et al.*, 2009 ▸).

**Table 2:** Thermochemical calculations for Equation (1)

Ligand *L*	Δ*H*° (kJ mol^−1^)	Δ*S*° (J mol^−1^ K^−1^)	Δ*G*° (kJ mol^−1^)
dmise	−6.5	27.6	−14.8
deise	−5.4	24.2	−12.7
diise	−65.1	23.8	−72.2
